# COordination of Standards in MetabOlomicS (COSMOS): facilitating integrated metabolomics data access

**DOI:** 10.1007/s11306-015-0810-y

**Published:** 2015-05-26

**Authors:** Reza M. Salek, Steffen Neumann, Daniel Schober, Jan Hummel, Kenny Billiau, Joachim Kopka, Elon Correa, Theo Reijmers, Antonio Rosato, Leonardo Tenori, Paola Turano, Silvia Marin, Catherine Deborde, Daniel Jacob, Dominique Rolin, Benjamin Dartigues, Pablo Conesa, Kenneth Haug, Philippe Rocca-Serra, Steve O’Hagan, Jie Hao, Michael van Vliet, Marko Sysi-Aho, Christian Ludwig, Jildau Bouwman, Marta Cascante, Timothy Ebbels, Julian L. Griffin, Annick Moing, Macha Nikolski, Matej Oresic, Susanna-Assunta Sansone, Mark R. Viant, Royston Goodacre, Ulrich L. Günther, Thomas Hankemeier, Claudio Luchinat, Dirk Walther, Christoph Steinbeck

**Affiliations:** 1 European Molecular Biology Laboratory, European Bioinformatics Institute (EMBL-EBI), Wellcome Trust Genome Campus, Hinxton, Cambridge CB10 1SD UK; 2 Department of Stress and Developmental Biology, Leibniz Institute of Plant Biochemistry, Weinberg 3, 06120 Halle, Germany; 3 Max Planck Institute of Molecular Plant Physiology, 14476 Potsdam-Golm, Germany; 4 Magnetic Resonance Center (CERM), University of Florence, 50019 Sesto Fiorentino, FI Italy; 5 Department of Biochemistry and Molecular Biology, Faculty of Biology, IBUB, Universitat de Barcelona, Diagonal 643, 08028 Barcelona, Spain; 6 INRA, Univ. Bordeaux, UMR1332 Fruit Biology and Pathology, Metabolome Facility of Bordeaux - MetaboHUB, Functional Genomics Center, IBVM, Centre INRA Bordeaux, 71 av Edouard Bourlaux, 33140 Villenave d’Ornon, France; 7 Centre of bioinformatics of Bordeaux (CBiB), University of Bordeaux, 33000 Bordeaux, France; 8 University of Oxford e-Research Centre, 7 Keble Road, Oxford, OX1 3QG UK; 9 University of Bordeaux, CBiB/LaBRI, 33000 Bordeaux, France; 10 School of Chemistry & Manchester Institute of Biotechnology, University of Manchester, 131 Princess St., Manchester, M1 7DN UK; 11 Microbiology & Systems Biology, TNO, Zeist, Netherlands; 12 Division of Analytical Biosciences, Leiden Academic Center for Drug Research, Leiden University, Leiden, Netherlands; 13 Zora Biosciences OY, 02150 Espoo, Finland; 14 Medical Research Council Human Nutrition Research, Fulbour Road, Cambridge, CB1 9NL UK; 15 Department of Biochemistry, University of Cambridge, Cambridge, CB2 1GA UK; 16 Computational and Systems Medicine, Department of Surgery and Cancer, Imperial College London, South Kensington, London, SW7 2AZ UK; 17 School of Cancer Sciences, University of Birmingham, Edgbaston, Birmingham, B15 2TT UK; 18 School of Biosciences, University of Birmingham, Edgbaston, Birmingham, B15 2TT UK; 19 FiorGen Foundation, 50019 Sesto Fiorentino, FI Italy

**Keywords:** Metabolomics, Metabonomics, Data standards, Data exchange, e-Infrastructure, Coordination and data sharing community

## Abstract

Metabolomics has become a crucial phenotyping technique in a range of research fields including medicine, the life sciences, biotechnology and the environmental sciences. This necessitates the transfer of experimental information between research groups, as well as potentially to publishers and funders. After the initial efforts of the metabolomics standards initiative, minimum reporting standards were proposed which included the concepts for metabolomics databases. Built by the community, standards and infrastructure for metabolomics are still needed to allow storage, exchange, comparison and re-utilization of metabolomics data. The Framework Programme 7 EU Initiative ‘coordination of standards in metabolomics’ (COSMOS) is developing a robust data infrastructure and exchange standards for metabolomics data and metadata. This is to support workflows for a broad range of metabolomics applications within the European metabolomics community and the wider metabolomics and biomedical communities’ participation. Here we announce our concepts and efforts asking for re-engagement of the metabolomics community, academics and industry, journal publishers, software and hardware vendors, as well as those interested in standardisation worldwide (addressing missing metabolomics ontologies, complex-metadata capturing and XML based open source data exchange format), to join and work towards updating and implementing metabolomics standards.

## Introduction

Metabolomics (Bundy et al. 2009; Clayton et al. 2006; Eckhart et al. 2012; Holmes et al. 2008)^1^ and fluxomics (metabolic flux analysis, Zamboni, Nicola et al. “13C-based metabolic flux analysis.” *Nature protocols* 4.6 (2009): 878–892) measurements mark the end point closest to the phenotype of organisms, reflecting changes in organisms influenced by external parameters such as nutritional, environmental or toxicological interactions. In this context, due to its dynamic nature, metabolomics is of considerable value for examples in personalised medicine, especially as it captures rapid responses close to the phenotype and in concert with the genome, transcriptome and epigenome (van der Greef et al. 2006, Nicholson et al. 2011). For such methods to succeed in a personalised medicine context, robust traceable standardisation is essential, covering storage and exchange of metabolomics and fluxomics data. Moreover, new applications that link *metabolomics and biobanks* are emerging: metabolomics may be used as an efficient tool to monitor the quality of stored samples and to establish the optimal standard operating procedures (SOPs) for the pre-analytical handling of bio-specimens (Bernini et al. 2011). Metabolomics is rapidly becoming an essential tool in the *screening of food products,* which is highly regulated and follows standard guidelines. Furthermore it is being investigated as a potentially transformative technology for the *screening of chemical safety*, not only for traditional industrial and domestic chemicals but also for the safety assessments of engineered nanomaterials as well as novel compounds generated through synthetic biology.

Considering the diversity and breadth of metabolomics applications, not forgetting complexity and diversity of the analytical technologies in use, there is a clearly identified need for standardisation that evolves with the technologies and is sufficiently inclusive to cover all metabolomics applications.

## What has been achieved so far in metabolomics standards

The momentum for metabolomics standards started in 2004–2005 with initiatives such as the standard metabolic reporting structure initiative or SMRS (Lindon et al. 2005) and the Architecture for Metabolomics consortium or Armet (Jenkins et al. 2004); these were mainly focused on an aspect of metabolomics standards, for example nuclear magnetic resonance (NMR) based metabonomics or plant-based metabolomics. There were several other initiatives at the time, however all efforts eventually resulted in the formation of the metabolomics standards initiative (MSI) in 2005 (Castle et al. 2006; Fiehn et al. 2006). This was focused on community-agreed minimum reporting standards and providing initial efforts on the descriptions of the experimental metadata describing a metabolomics study. This culminated in a series of manuscripts published in 2007 that considered all the components undertaken in metabolomics experiments (Sansone et al. 2007; Fiehn et al. 2007; Hardy and Taylor 2007) summarized in (Goodacre 2014). One major outcome was the formation of five different working groups (WG) to consider each aspect of the metabolomic pipeline; biological context metadata WG, chemical analysis WG, data processing WG, ontology WG and exchange format WG, with the task of collecting relevant metabolomics standards and a forum for discussion (Goodacre et al. 2007; Morrison et al. 2007; Rubtsov et al. 2007; Sumner et al. 2007; Werf et al. 2007). However, there have been limited practical applications for such descriptions, with some exceptions (Ludwig et al. 2012; Bais et al. 2010; Ferry-Dumazet et al. 2011; Griffin et al. 2011; Scholz and Fiehn 2007), in part owing to a lack of tools to facilitate implementation or a widely used database to enforce such standards. Most projects or databases focused on one particular technology or limited to a particular species or type of analytical technique. In order to implement agreed and acceptable guidelines on reporting identified metabolites, an application platform such as database i.e. a metabolomics repository in addition to a journal publication is required. 2012 saw the release of MetaboLights (http://www.ebi.ac.uk/metabolights), the first general purpose database in metabolomics, developed and maintained by the European Bioinformatics Institute (EMBL-EBI), one of the largest open access data providers in the world (Haug et al. 2013; Salek et al. 2013a). MetaboLights combines small molecule ‘reference’ layer with information about individual metabolites, their chemistry, spectrometry and biological roles with a study archive, where primary data and metadata from metabolomics studies are ontologically tagged and stored. Such depositions receive a stable identifier for each study, which can be quoted in related publications and can be used to access the data long term. Making metabolomics data publicly accessible allows it to justify researchers’ findings in a peer-reviewed publication, increases the possibility of wider collaborations within the metabolomics community and ultimately gives a study higher visibility and increased citation (Nature Genetics 2009). MetaboLights adheres to MSI standards and uses the Investigation/Study/Assay (ISA) tab-delimited format (Rocca-Serra et al. 2010), which makes it interoperable with a large number of other ongoing projects dealing with biological study data, including other ‘omics datasets. Specific scientific fields have developed their own systems biology solutions (e.g. dbNP developed by NuGO, covering the Nutritional Phenotype), and the metabolomics data of such sites should be made exchangeable with other metabolomics databases (for instance by implementing an export to ISA-Tab format for the study metadata as well as exporting the results).

## COSMOS: the way forward in standards

The FP7 EU Initiative ‘coordination of standards in metabolomics’ (COSMOS) brings together leading vendors, researchers and bioinformaticians of the European metabolomics community, members of the MSI, members of the international Metabolomics Society, along with other stakeholders worldwide. One of the COSMOS initiative goals is to develop a robust data infrastructure for metabolomics data and metadata representation and exchange in order to support workflows for a broad range of metabolomics applications (Salek et al. 2013b; Steinbeck et al. 2012). The potential of metabolomics cannot be harvested without major standardisation of formats and terminologies, therefore we leverage on and extend earlier efforts initiated by the MSI and currently operating under the Metabolomics Society, in part via that society’s dedicated Data Standards task group. As is the case for other high-throughput “-omics” disciplines, metabolomics is seeing a paradigm shift from hypothesis-driven to data-driven science (Cox and Mann 2011; Goodacre et al. 2004). As a result, metabolomics data are constantly growing with a plethora of analysis tools. Cross-site data comparison remains a challenging task due to the different access modalities for the different local repositories. Hence, currently the generated data often ends up in data silos or worse as data dumps or ‘data-graveyards’. This situation constitutes a need for the establishment of open data standards and accessible repositories that allow researchers to store, exchange and compare metabolomics data with pertinent metadata information, and thus communicate on a scientific level without getting stuck in vendor specific data formats. As different scientific fields continue to develop their own specific solutions, due to specific analytical solutions or meta-data requirements, COSMOS will invite these fields to adhere to the general metabolomics standards and export to the metabolomics solutions developed by COSMOS. To compare data between labs, the data needs to be stored in a way that allows concise objective interpretation and reproducibility, i.e. the type, origin and treatment of samples and corresponding spectra needs to be described in an unambiguous manner using a common communication channel. Here, controlled vocabularies (CVs) and ontologies can be used to standardise the terminology used to represent scientific facts, e.g. tissue or fluid description, sample storage, preparation and analysis conditions. Another benefit of CVs are their knowledge representation capabilities, i.e. their taxonomic backbone that can be exploited to gather more subsumptive (a more general/abstract) or a more excluding, search specific attributes.

To work out commonly agreed-upon metabolomics data standards, the COSMOS initiative coordinates with metabolomics and bioinformatics experts to work on open data exchange formats (syntax) and data semantics that maximize interoperability with other omics standards (Nature Genetics 2012). This is achieved among other solutions, by using (i) the general-purpose Investigation/Study/Assay tabular format or ISA-Tab (Rocca-Serra et al. 2010) for the experimental information and (ii) adapting the XML-based formats for the instrument-derived “raw” data types by the proteomics standards initiative (PSI) (Orchard et al. 2003b; Orchard et al. 2003a), e.g. *mzML* (Martens et al. 2011). Data completeness can then be verified using validator software enforcing minimum information recommendations such as the MSI Core Information for Metabolomics Reporting (CIMR; http://biosharing.org/bsg-000175) (Sumner et al. 2007). The standardisation efforts in COSMOS for nuclear magnetic resonance spectroscopy (NMR) and mass spectrometry (MS) data, with potential to encompass new and alternative technologies as they are developed, and supporting tools, will form the basis for funders and publishers to recommend data deposition. The submitted dataset, in repositories such as MetaboLights (Haug et al. 2013) or the Netherlands Metabolomics Centre (NMC) Data Support Platform (DSP) could then be used to justify findings in a publication. Unlike in other -omics domains, as for example ArrayExpress (Parkinson et al. 2009) and Gene Expression Omnibus GEO (Barrett et al. 2013) for transcriptomics, the previous lack of such open, centralised and persistent data deposition repositories in the metabolomics field has been criticised by journal editors who face the tedious task of having to judge whether conclusions based upon megavariate data are sound and justified. Here open-access repositories using our standards, data curation and capture tools, such as the ISA software suite (Rocca-Serra et al. 2010), as well as others, will facilitate curation and storage of the metadata at the source, and streamline submission to MetaboLights. A growing number of data publication journals, e.g. BioMedCentral’s GigaScience^2^ and Nature Publishing Group’s *Scientific Data* 3 now support the ISA format for supplementary experimental data and as a means to capture metadata descriptions. ISA-Tab format is currently in use and supported by public data repositories such EMBL-EBI Metabolights (accounting for about 200 datasets, 90 of which are currently publicly available), but also several major european toxicogenomics projects (Carcinogenomics,^4^ DiXa^5^ and InnoMed PredTox and ToxBank^6^). These projects fully exploit the capability of the ISA-Tab format to support an array of assay type allowing to recording multi-omics assays. Furthermore, ISA developers have a range of tools for converting from various sources (ArrayExpress,^7^ SRA^8^) into ISA-Tab format.

Ultimately, we hope that COSMOS will help experts in NMR spectroscopy and MS-based metabolomics to communicate their results in a more objective comprehensive, persistent and efficient way, and spanning and integrating multiple domains such as medical, environmental, plant and food sciences.

Although funding by the European Community is limited to a number of European expert scientists, COSMOS links to major initiatives world-wide. For example with the US National Institutes of Health (NIH) Common Funds Metabolomics Initiative (http://commonfund.nih.gov/Metabolomics/) which has awarded funding for six Regional Comprehensive Metabolomics Research Cores (RCMRC), and a Data Repository and Coordination Centre (DRCC), to act as a North American hub for metabolomics related research.^9^ COSMOS also reaches and establishes links with other related e-infrastructures initiative such as the new European-wide ELIXIR project,^10^ Biobanking and Biomolecular Resources Research Infrastructure^11^ (BBMRI) via BioMedbridges^12^ consortium, Human Metabolome Database (HMDB) in Canada (Wishart et al. 2013), Platform for RIKEN Metabolomics (PRIMe) in Japan (Sakurai et al. 2013) and Beijing Genomics Institute (BGI)^13^ in China.

### Metabolomics data exchange standards

The COSMOS work on standardisation aims to build on the foundational work by PSI and MSI and further develop and contribute to data exchange formats, ranging from raw data in MS and NMR, the reporting of metabolite quantification and metabolite identification, to the experimental metadata. We aim to extend the open standards for MS data exchange initiated by PSI, such as mzML (Martens et al. 2011), mzIdentML (Jones et al. 2012) and mzQuantML (Walzer et al. 2013) to meet the requirement of metabolomics experiments for reporting MS experiments. One example are GC–MS based metabolomics experiments, where data are often available in either a closed vendor format or as netCDF, where the latter provides only very few metadata acquisition parameters and fails to capture advanced MS experiments such as GC × GC–MS or tandem-MS with GC–MS instruments. This requirement led us to augment the PSI-MS controlled vocabulary with GC specific terms and concepts, which have already been included in the current PSI-MS ontology. To avoid a chicken-and-egg problem, we have collected raw data examples “in the wild”; and checked which GC–MS vendor formats can be converted to mzML. Currently, file formats by Agilent Technologies, Bruker Biosciences Corporation, Waters Corporation and Thermo Fisher Scientific are companies that their file format is readily supported by the Proteowizard Open Source converter (Kessner et al. 2008; Chambers et al. 2012). Other companies, such as LECO Corporation and Bruker Biosciences in addition have software to export their file format to mzML. On the consumer side, we surveyed which mzML parsing libraries are available for the community. Parsers for mzML exist for the languages C++, Java, R and Python, which should cover the majority of the current software developments in the metabolomics community, and we shall of course supplement to these as necessary. Therefore, mzML can be a strong suggestion or even a requirement for data deposition in public repositories. Formats that capture metabolite identification and reporting of quantification results also need to be adapted for MS metabolomics experiments, and require real-world tests, software support and community adoption. We hope to make COSMOS a platform for community engagement with adaptation and development of these formats to suit the metabolomics community needs, and as mentioned above we regularly consult with the Metabolomics Society. In addition, COSMOS developing the missing XML exchange formats for NMR spectroscopy such as nmrML, nmrIdentML, nmrQuantML and nmrTab needed by databases and open source software such as NMRLab/MetaboLab (Ludwig and Gunther 2011) Bayesian AuTomated Metabolite Analyser for NMR spectra (BATMAN) (Hao et al. 2012) and rNMR (Lewis et al. 2009). These developments take place on www.nmrml.org and https://github.com/nmrML/ and include the XML schema, controlled vocabulary (Schober et al. 2014), example files, and reader, writer, conversion and validation software. As part of this approach we have begun and will continue to interact with the wider community to ensure wide adoption and call for implementations of the standards during the design phase, which helps to catch design errors before the standard is published. With semantic web technology in mind, these standards will pave the way for metabolomics data to be part of the world of linked (and open) data (Murray-Rust 2008). Preliminary work in currently underway, leveraging work by the ISAteam in the field of linked data to offer MetaboLights metadata content as linked data (González-Beltrán et al. 2014a).

### Metabolomics databases and repositories

The power of broad, system-wide -omics relies on the potential to interrogate datasets from new perspectives. Researchers not involved in the original data generation process may reuse data differently from the original purpose that motivated the data collection. Unleashing this potential also in the heterogeneous metabolomics landscape requires the availability of metabolite level (ideally quantified) and profile data along with adequate metadata. Therefore, the COSMOS consortium is committed to develop the MetaboLights database further as a centralised data exchange and storage platform. MetaboLights will serve as a common publication hub and make it possible to connect different resources while keeping the data interoperable (e.g. connect to data in other resources, such as the NMC-DSP (van Ommen et al. 2010)). In the metabolomics field, a large number of custom and often technology focused, substance-class, or species-centric databases exist and are continuously developed for example; the Golm Metabolome Database or GMD (Hummel et al. 2010) LipidMaps (Fahy et al. 2009), PlantMetabolomics.org (Bais et al. 2010) and MeRy-B (Ferry-Dumazet et al. 2011). Defining a sensible balance of centralised versus decentralised information storage can be resolved by developing and applying standards and exchange formats. Also, buy-in from users as well as publishers will have to be achieved. The journal *Metabolomics*, which is the official journal of the Metabolomics Society, has since 2010 encouraged authors to ensure their papers are as MSI compliant as possible (Goodacre 2010) and is committed to supporting the COSMOS consortium in its endeavours for metabolomics standardisation. This ethos is also being adopted by other journals including *Metabolite, EMBO* and others to join.

Integrative analysis of datasets is essential in order to achieve better understanding of phenotypes. Moreover, interfacing with dedicated databases utilising metadata annotation tools will engage and enable a broad user base to export data from their local systems into ISA-Tab formatted data sets, and subsequently to easily import or submit to *MetaboLights*. MetaboLights and the ISA team have been working on implementing principled curation guidelines, ensuring consistency in the reporting of experimental designs. As a machine readable XML dialect, the schema based XEML (Hannemann et al. 2009) provides means to store experimental design and metadata describing the actual experiment, together with links to one or more independent databases hosting the actual experimental results as well as export the results in ISA-Tab format. The XEML-Lab sources and binaries for different operating systems can be accessed and downloaded from https://github.com/cbib/XEML-Lab.

In addition to the obvious breadth of experimental conditions, the diversity of laboratory specific SOPs, even within the most commonly employed measurement techniques such as NMR and MS, renders the joint interpretation of data produced by different labs difficult. Hence, standardised and machine-readable metadata describing all aspect of experimental conditions are an essential prerequisite to allow a quick, objective and hence meaningful selection of experiments suitable for comparison. In a single-user environment, experimental metadata annotation can be efficiently handled using the ISAcreator, part of the ISA software suite. In addition, the COSMOS consortium also aims to develop standards to connect to existing specialised databases such as the GMD, MeRy-B, the NMC-DSP/dbNP, as well as other similar resources using alternative metadata annotation tools such as XEML (Hannemann et al. 2009) or the automatic processing pipelines within Bioconductor packages (Gonzalez-Beltran et at. 2014b) and Bioportal powered ontology (Maguire et al. 2013).

### Data deposition workflow

**Fig. 1:**
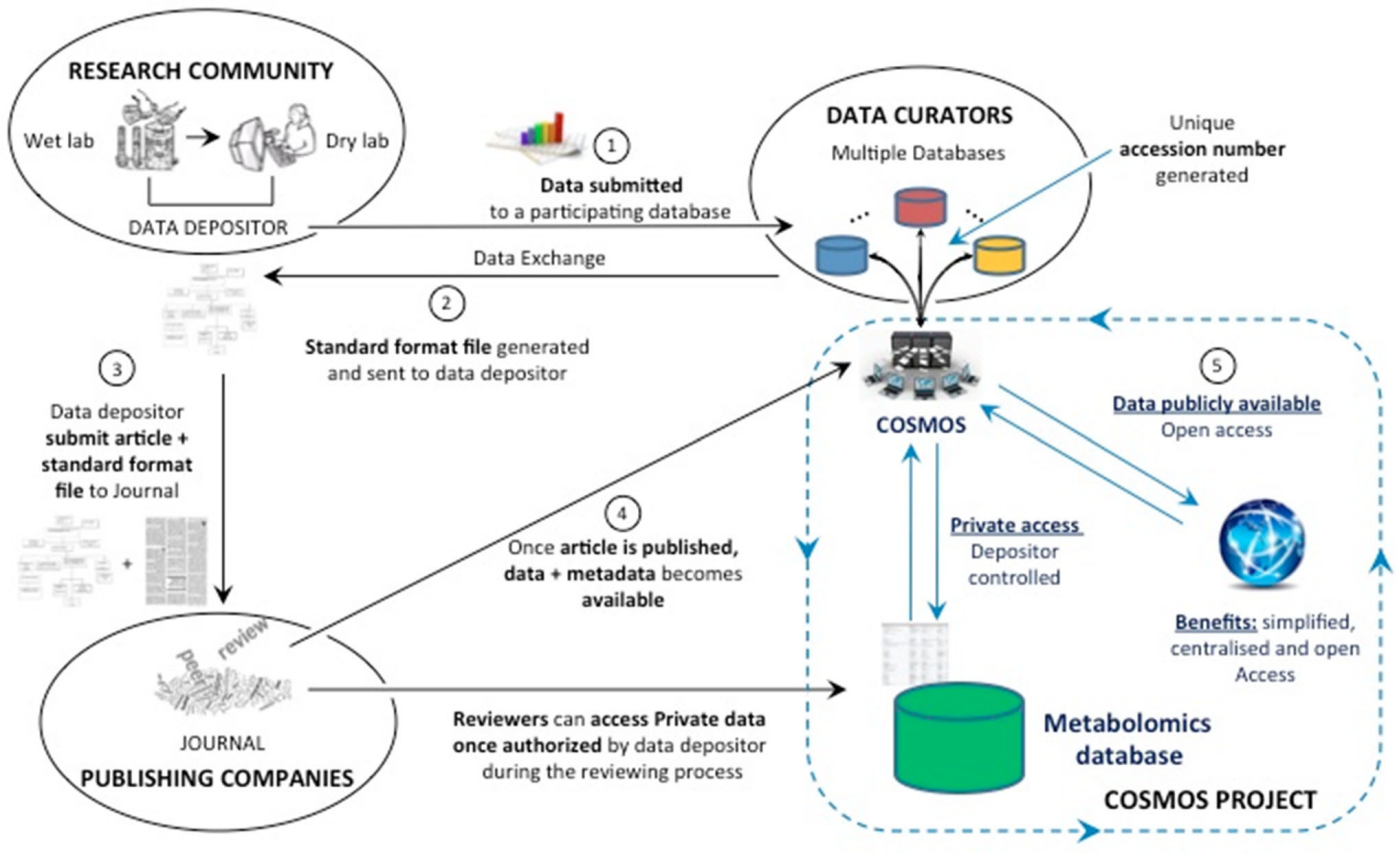
Initial model for the COSMOS data deposition workflow system. The data deposition cycle is initiated when a Submitter (who has generated or owns the study material) submits his/her metabolomics study to a specific associated database (*1*). Once the data submission has been completed, fulfilling the requirement of the associated repository submission guideline, a unique COSMOS accession number will be generated. The COSMOS engine will then properly annotate, format and store the minimum agreed metadata according to proposed reporting standards suggested by COSMOS partners (*2*, *3*). COSMOS will bring together publishers and other metabolomics repositories to come to final agreement on a data workflow specifying minimum metadata exchange, associated raw data, source code and any additional information that can be shared (*4*, *5*). Open access to the system will ensure that any interested party can benefit from the standardized resources

**Fig. 2:**
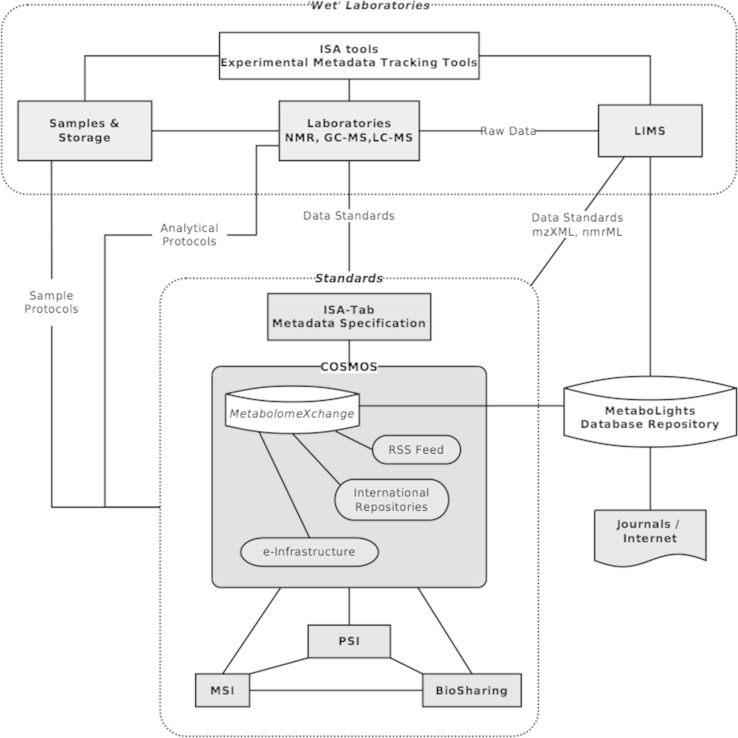
COSMOS initiative workflow. Overview of COSMOS initiative role in metabolomics standards, databases, data exchange and dissemination of metabolomics experiments. *Green* metabolomics labs experimental workflow from lab based data generation, metadata collection to interaction with LIMS systems. *Pink* standardisation initiative and minimum information reporting agreement involved or used within the COSMOS project. *Blue* Dissemination and role of journal and link to other e-infrastructures. *White* databases and tools used to capture experimental data and metadata (Color figure online)

Making raw data available to the interested research community has clear benefits to the transparency and trustworthiness of those scientific studies. Scientists might choose a variety of resources for their data deposition, depending on their preferred technology. A comprehensive workflow should protect data proprietary interests, security (data will not be made publicly available until the associated publication has a bona fide DOI or the authors request immediate data release), and confidentiality as required. To ensure proper reporting of metabolomics data and metadata (Salek et al. 2013c), COSMOS will set clear procedures for data submission and deposition, as well as metabolomics results reporting considering publishing requirements. These will be in line with the existing MSI guidelines. These guidelines are currently being carefully discussed, elaborated and agreed by all COSMOS partners. COSMOS is also taking every opportunity to engage fully with stakeholders and potential collaborators on planning, discussion and implementation of the guidelines for data deposition workflow. Careful planning of the data deposition flow, its control policies and actions, will ensure that the utility of the system is maximized and quality-controlled for use inside and outside Europe. A proposed model for the data deposition workflow, drafted from discussions within COSMOS, is shown in Fig. 1. The workflow definition will prioritize simplicity, usability, annotation quality, the plurality of metabolomics resources and databases, to ensure connectivity between similar studies and to provide rapid matching results to the end users. We envisage that in the future additional purpose-built databases will be created that can potentially be integrated into the proposed workflow. This will include MetaboLights and the NIH funded Metabolomics Workbench.^14^ The first phase of the data deposition cycle is temporary and all data and associated information are kept private. Once the study has been officially published and the depositor agrees to share the data (making it open access), the COSMOS “metabolomeXchange” system will automatically announce and broadcast availability of such studies to the broad research community (Fig. 2). In addition to the minimum required metadata (e.g. accession, title, abstract, publication date, URL and submitters name), the COSMOS “metabolomeXchange” system allows datasets to be annotated using additional metadata information. This would enable the metabolomics community (both metabolomics researchers and databases) to query efficiently and readily identify interesting and reusable metabolomics data sets.

The default for most journals will be to promote open access of the data as soon as the study is published, while the accepted academic standard is to allow others to access the work. All parties involved will benefit from sharing raw data, processed data, metadata, statistical methods and source codes. By increasing the visibility of their work, depositors are likely to boost citations. Publishers and journals will expose their publications to a greater number of potential readers and enhance the overall impact of the work. In addition, through COSMOS the research community will gain free access to a vast amount of well documented and easy to access scientific information.

### Coordination with BioMedBridges and biomedical ESFRI infrastructures

COSMOS aims at building a network of close interactions with the European biomedical infrastructures. A particular interest is in the infrastructures for which metabolomics is most relevant such as BBMRI, ELIXIR, EU-Openscreen,^15^ EuroBioimaging^16^ and EURODISH,^17^ all of which are participating in the EC-funded project BioMedBridges.^18^ Our main objective is to obtain indicators, useful to prioritize various COSMOS activities, in order to obtain effectively responses needed in large-scale EU biomedical infrastructures. BioMedBridges and COSMOS are reasonably complementary as they address different levels of information—COSMOS is more focused on the experimental side whereas BioMedBridges tackles the higher complexity of human disease, investigated by a plethora of different technologies, of which one is metabolomics. Another important collaboration is COSMOS with BBMRI. Human biobanks are structured resources that store: (a) human biological materials and/or information generated from the analysis of the same and (b) extensive associated information. An emerging aspect would be the usage of metabolomics as an efficient tool to monitor pre-analytical sample variations as metabolites are known to be prone to degradation phenomena, possibly more so than other clinical biomarkers (e.g., DNA, RNA, proteins). Another application of metabolomic profiling in relation to biobanking is in assessment of quality and usage history for samples and their collection, handling, traceability and storage. Here COSMOS may play a key role in the development of relevant required standardised formats. Formal interactions between COSMOS and BBMRI are being established to coordinate efforts: first, the coordinator of BBMRI, has been nominated in the Advisory Board of COSMOS; second, and also within the frame of BBMRI (which specifically aims at integrating existing biomolecular resources, technologies, standards and know-how), the University of Florence Magnetic Resonance Center (CERM/CIRMMP) partner of COSMOS is establishing a European multi-site Expert Center on metabolomics.

### Outreach and dissemination

We will maintain a close link between the COSMOS consortium and the wider metabolomics and biomedical communities, as well as other related scientific fields. We shall continue to use metabolomics workshops, meetings and conferences to interact with the wider communities, metabolomics or others, to get all parties involved with the initiative. In addition, we will use publications and social media, news articles and blogs to raise awareness of metabolomics standards and the services provided by the COSMOS consortium. This will be from data submission to providing support on different views on how metabolomics data may be reported. There is a great need for increasingly stringent requirements for data quality that is publicly available to the whole metabolomics community. Through the existing framework of the Metabolomics Society, we will ensure broad community input into the services and standards for metabolomics data representation developed by COSMOS consortium, and it may come as no surprise that several of the authors have served on one or more of the Boards of the Metabolomics Society.

## Concluding remarks

COSMOS will implement harmonised and compatible data deposition strategies and contribute to annotation workflows, providing data producers involved in metabolomics experiments with a single point of submission, while allowing other data entry points through facilitation of interoperability. The data deposition and exchange workflow in the COSMOS consortium will be formally defined, agreed, and documented in relation with MetaboLights and all partnering databases in Europe and worldwide that would like to participate, and we welcome discussions from other data providers and software houses. A guideline for submitted data will be generated and COSMOS will strive to make sure that all metabolomics data submitted to partner databases are exchangeable with this standard. Since the adoption of minimal standards for metabolomics by the relevant journals is a major goal of this coordination action, we will consult with journal publishers and ensure data annotation quality and consistency, according to the required standard level set by each journal. For example, collaboration with data journals such as *GigaScience* and Nature’s *Scientific Data* will be streamlined, given these journals already use the ISA-Tab format for data submission. We are also working closely with *Metabolomics,* which is the official journal of the Metabolomics Society.

The COSMOS consortium ultimately develops the standards and infrastructure for—and with—the metabolomics and fluxomics community. For the most efficient interaction we have already, and will continue, to organise stakeholder meetings as satellite events to major metabolomics meetings, individual staff exchange between partners, as well as larger workshops. These efforts will directly enable the implementation of COSMOS important deliverable—that of a robust data infrastructure and mechanisms for standards metabolomics data representation and data/meta-data exchange that will enrich metabolomics science.

## Footnotes

1 "Metabolome | Metabolomics Definition | InTechOpen." 2012. 20 Aug. 2013 http://www.intechopen.com/books/metabolomics.

2 "GigaScience." 2011. 20 Aug. 2013 http://www.gigasciencejournal.com/.

3 "Scientific Data - Nature." 2013. 20 Aug. 2013 http://www.nature.com/scientificdata/.

4 Vinken "The carcinoGENOMICS project: critical selection of model …" 2008. http://www.ncbi.nlm.nih.gov/pubmed/18514569.

5 "diXa." 2012. 12 Mar. 2015 http://www.dixa-fp7.eu/.

6 "ToxBank | Data warehouse for toxicity data management." 2010. 12 Mar. 2015 http://toxbank.net/.

7 "ArrayExpress < EMBL-EBI - European Bioinformatics Institute." 2008. 12 Mar. 2015 https://www.ebi.ac.uk/arrayexpress/.

8 "Home - SRA - NCBI." 2009. 12 Mar. 2015 <http://www.ncbi.nlm.nih.gov/sra>.

9 "NIH announces new program in metabolomics." 2012. 20 Aug. 2013 http://www.nih.gov/news/health/sep2012/od-19.htm.

10 "home | ELIXIR." 2007. 20 Aug. 2013 http://www.elixir-europe.org/.

11 "BBMRI: Home." 2008. 20 Aug. 2013 http://www.bbmri.eu/.

12 "BioMedBridges: Home." 2012. 20 Aug. 2013 http://www.biomedbridges.eu/.

13 "BGI." 2009. 20 Aug. 2013 http://www.genomics.cn/.

14 "Metabolomics Workbench." 2013. 20 Aug. 2013 <http://www.metabolomicsworkbench.org/>.

15 "WELCOME TO EU-OPENSCREEN." 2009. 20 Aug. 2013 http://www.eu-openscreen.de/.

16 "Euro-BioImaging: Research Infrastructure for Imaging Technologies …" 2009. 20 Aug. 2013 http://www.eurobioimaging.eu/.

17 "EuroDISH: Home." 2013. 18 Sep. 2013 http://www.eurodish.eu/.

18 "BioMedBridges: Home." 2012. 20 Aug. 2013 http://www.biomedbridges.eu/.
